# Direct Intranodal Visualization During EBUS‐TBNA Using the Iriscope: A Novel Case Series

**DOI:** 10.1002/rcr2.70330

**Published:** 2025-09-14

**Authors:** Sammy Onyancha, Nesrin Tekeli, Emilia Nitsch, Birol Dedeoglu, Amel Havkic, Kati Kiil, Gernot Rohde

**Affiliations:** ^1^ Department of Pulmonology St. Elisabethen Krankenhaus Frankfurt Germany; ^2^ Dr. Senckenbergisches Institut für Pathologie Frankfurt am Main Germany; ^3^ Department of Respiratory Medicine Universitätsklinikum Marburg Marburg Germany

**Keywords:** EBUS, EBUS‐TBNA, iriscope, lung cancer staging, lymph node visualisation

## Abstract

Endobronchial ultrasound (EBUS)‐guided biopsy remains the gold standard in the diagnosis of mediastinal pathologies. However, visualisation of intranodal architecture has yet to be achieved. With the implementation of miniature video probes in interventional pulmonology, these tools may help bring an added dimension to EBUS‐guided diagnostics by allowing visualisation of intranodal architecture. We report a four‐patient case series demonstrating the clinical use of the Iriscope, a miniature videoprobe, for direct visualisation of mediastinal lymph nodes during EBUS‐guided biopsy. In each case, the Iriscope was deployed over a guide‐sheath through the working channel of the EBUS bronchoscope following transbronchial needle aspiration (TBNA) and inserted through the biopsy tract into the lymph node under continuous ultrasound guidance. Real‐time video imaging allowed inspection of nodal architecture and tissue consistency. The procedure was well tolerated in all cases, and no immediate complications were observed. This novel application introduces a new diagnostic dimension, potentially enhancing interpretation beyond cytologic or histologic sampling. All procedures were technically successful and complication‐free, supporting the feasibility and safety of this approach. These findings lay the groundwork for broader clinical validation and integration of direct intranodal endoscopy in interventional pulmonology.

## Graphical abstract

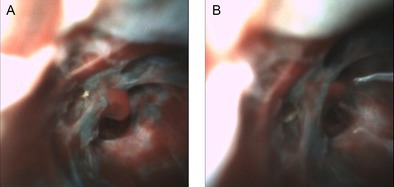

## 1 Introduction

Endobronchial ultrasound‐guided transbronchial needle aspiration (EBUS‐TBNA) has become the cornerstone in the diagnosis and staging of mediastinal lymphadenopathy, especially in lung cancer [1]. While the diagnostic yield of EBUS‐TBNA is well established, it is limited by reliance on cytologic or histologic specimens without direct visualisation of nodal architecture.

The Iriscope (Lys Medical, Belgium) is a 1.35 mm diameter flexible videoprobe designed for real‐time endoscopic imaging in confined anatomical spaces [2, 3, 4, 5]. Although previously validated in peripheral pulmonary applications, its intranodal use during EBUS procedures has not been reported. Here, we present the clinical application of the Iriscope for direct visualisation of mediastinal lymph nodes in four patients undergoing EBUS‐TBNA.

## 2 Case Series

Four patients underwent EBUS examination due to mediastinal lymphadenopathy and/or suspicion of malignancy. Lymph node sampling was done using a 22G crown‐tip TBNA needle. Following EBUS‐TBNA, the crown‐tip needle was used to create a tract into the lymph nodes, following the principles of the Ariza‐Pallares method of mediastinal cryobiopsy [6]. Iriscope insertion into the lymph nodes was done under sonographic guidance.

### 2.1 Case 1

**FIGURE 1:**
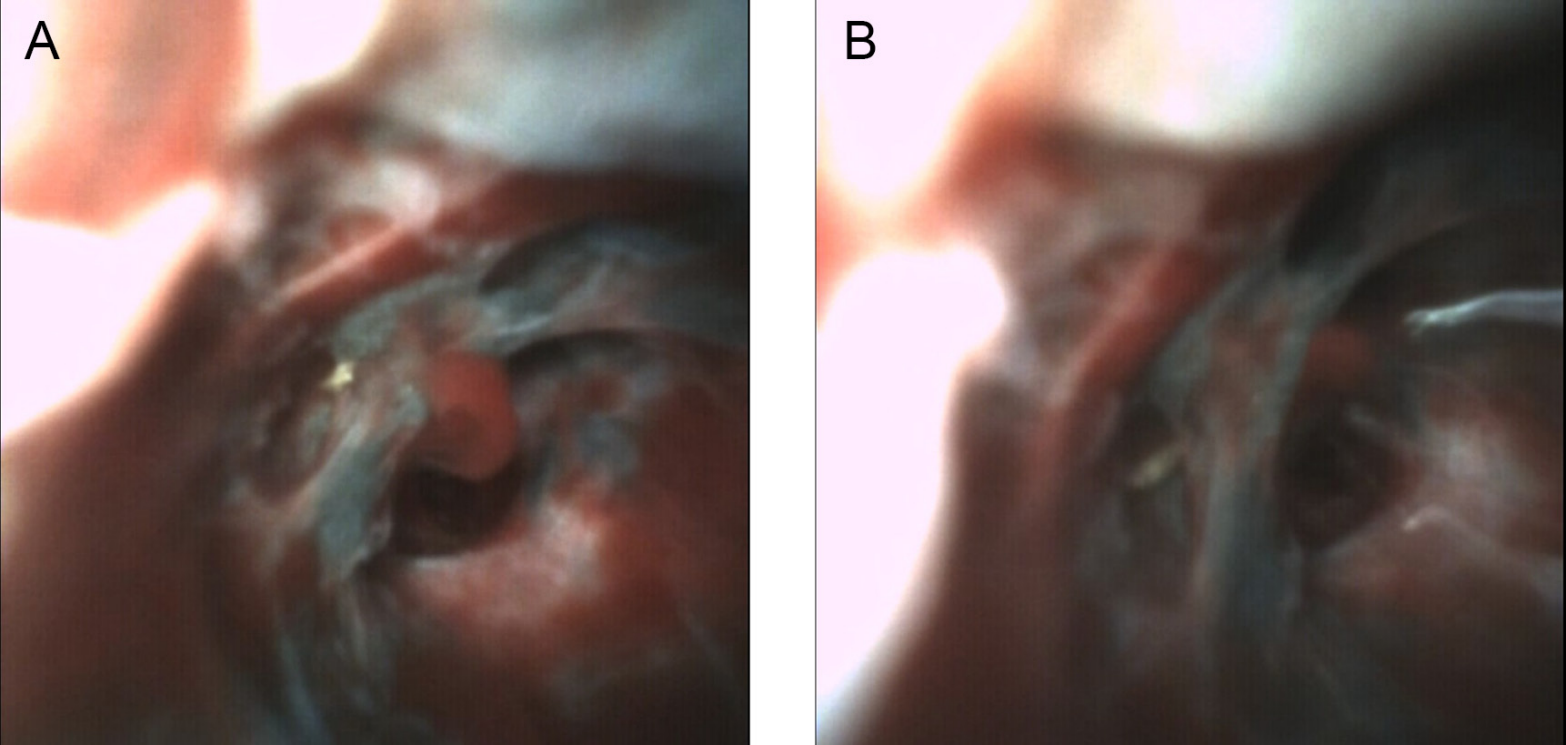
(A) and (B) Anthracotic lymph node parenchyma with white nodular foci.

A 62‐year‐old male with a right upper lobe mass and mediastinal lymphadenopathy in 4R, 4L as well as 11R underwent EBUS‐TBNA. A 22G crown‐tip needle was used for sampling and to create a tract into the lymph nodes. The Iriscope and guide sheath were advanced through the EBUS scope and into the nodes, revealing heterogeneous parenchyma with discrete white nodular foci in station 4R (Figure 1A,B). Histology confirmed adenocarcinoma in paratracheal nodes; hilar nodes were benign. The procedure was complication‐free.

### 2.2 Case 2

**FIGURE 2:**
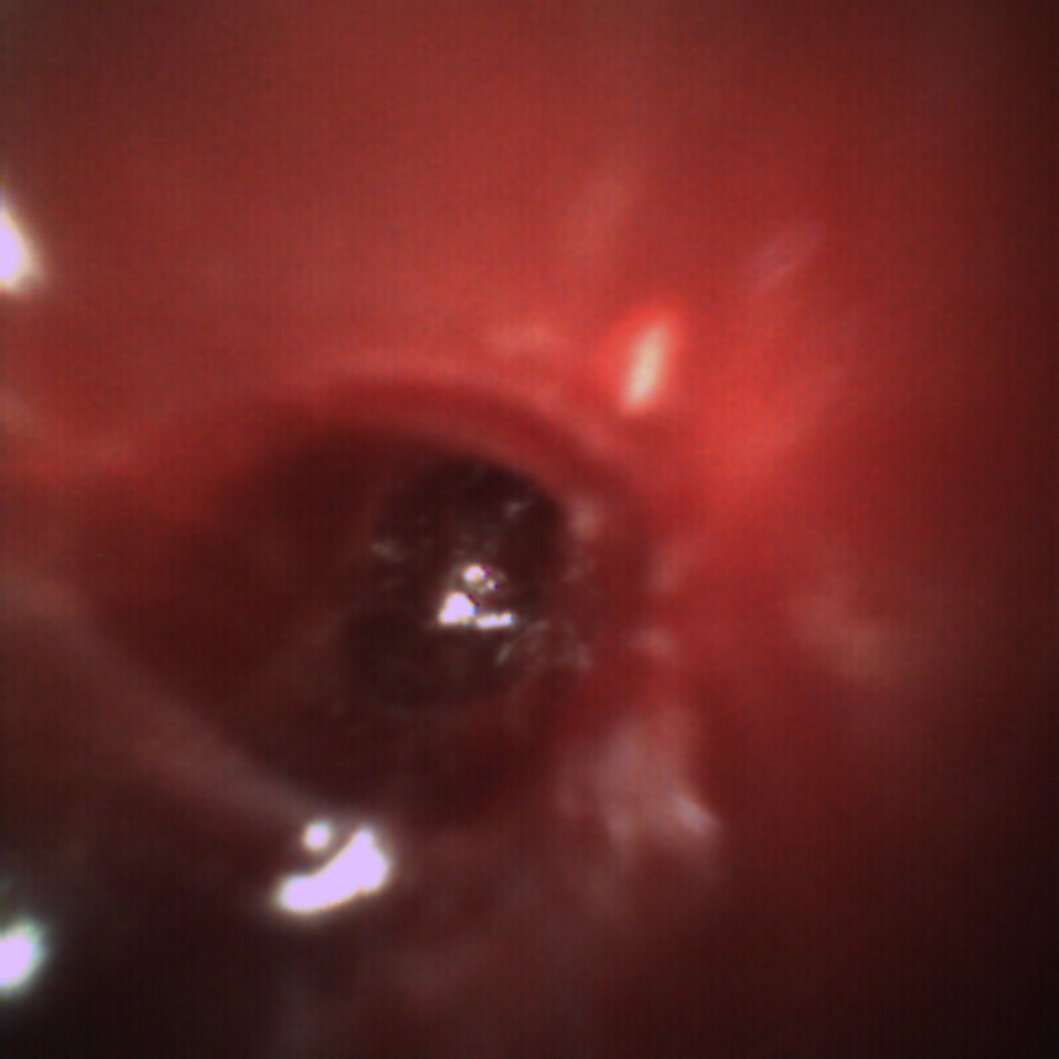
Lymph node parenchyma with uniform architecture.

**FIGURE 3:**
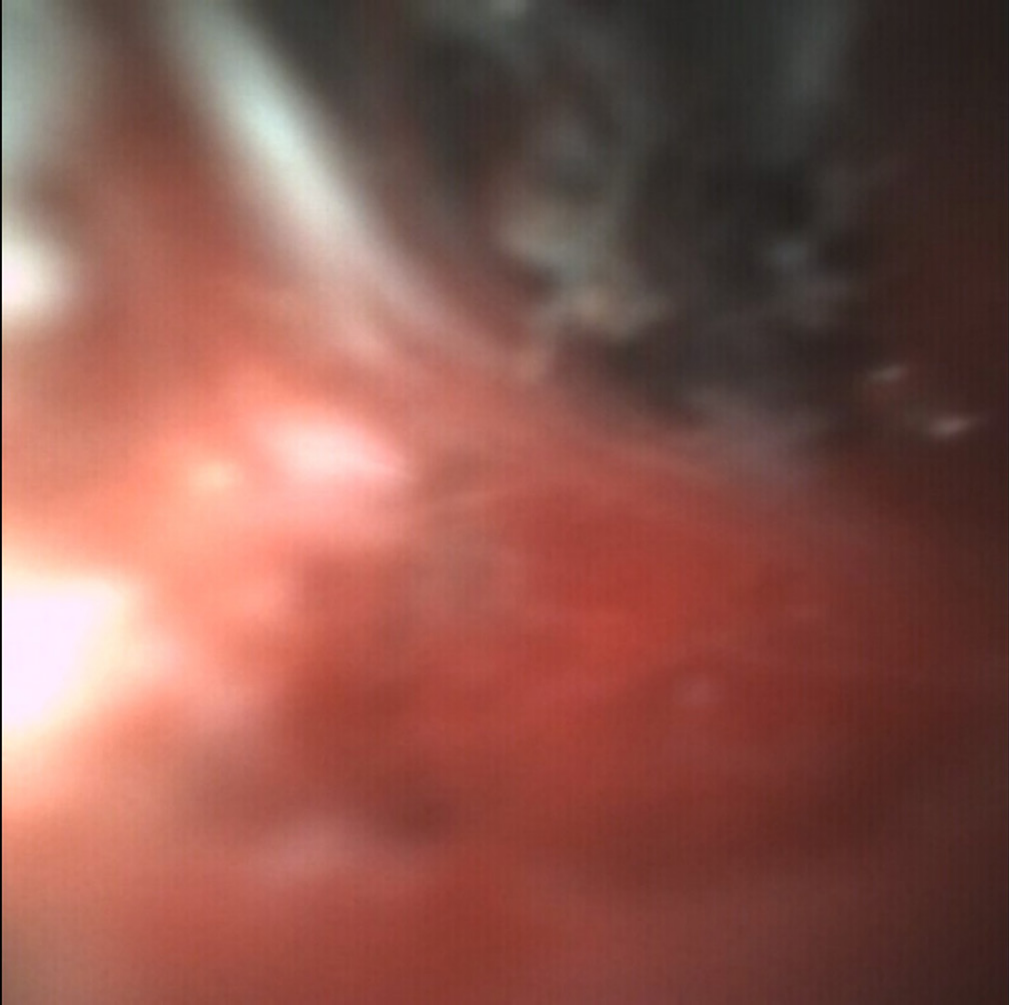
Lymph node parenchyma with scattered dark grey pigmentation.

A 68‐year‐old female with a history of breast cancer presented with PET‐positive mediastinal nodes in 7 and 4L. EBUS‐TBNA was performed using a 22G crown tip, followed by Iriscope insertion. Intranodal imaging showed uniform architecture in station 4L (Figure 2), (Video 1) and discrete scattered dark grey pigmentation in station 7 (Figure 3). Histological analysis revealed granulomatous inflammation, consistent with sarcoidosis. No adverse events occurred.

**VIDEO 1:** Iriscope guided intranodal lymph node visualisation for Case 2. Video content can be viewed at https://onlinelibrary.wiley.com/doi/10.1002/rcr2.70330.

### 2.3 Case 3

**FIGURE 4:**
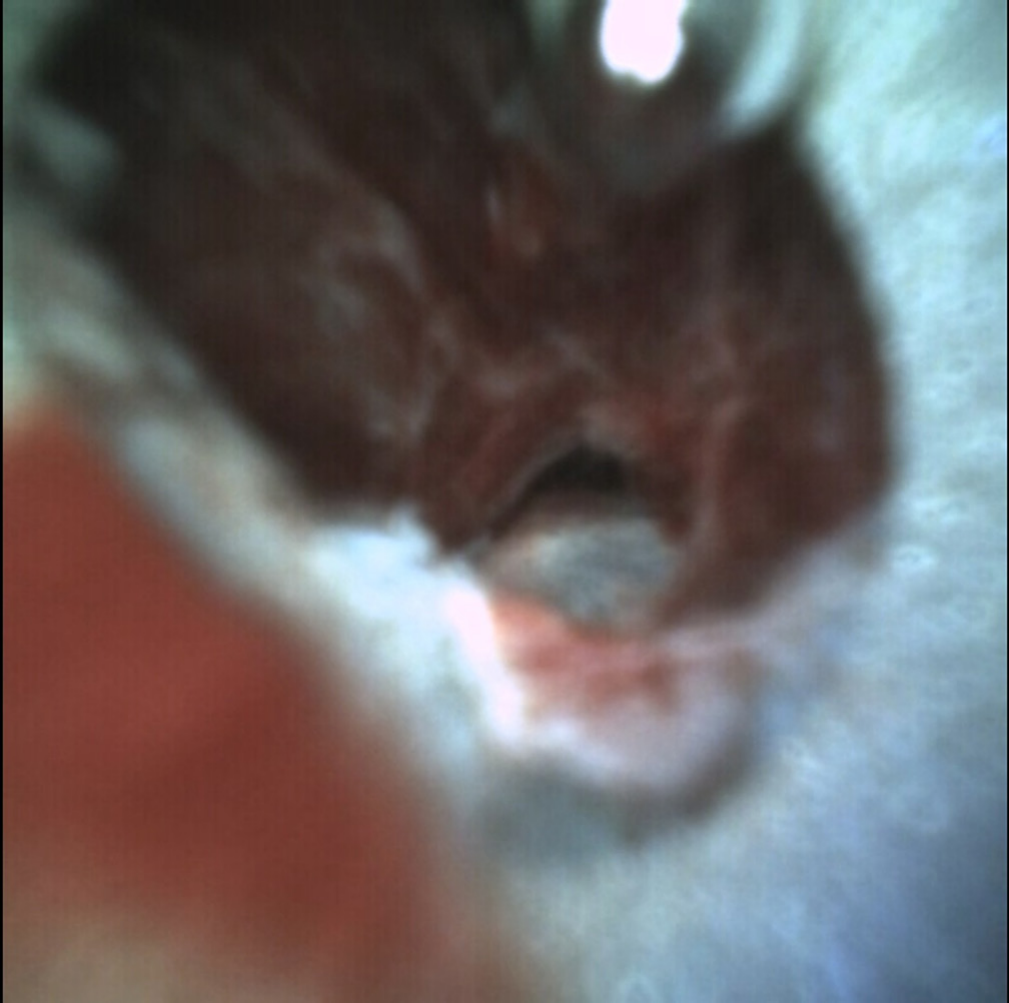
Lymph node parenchyma with diffuse white discoloration.

**FIGURE 5:**
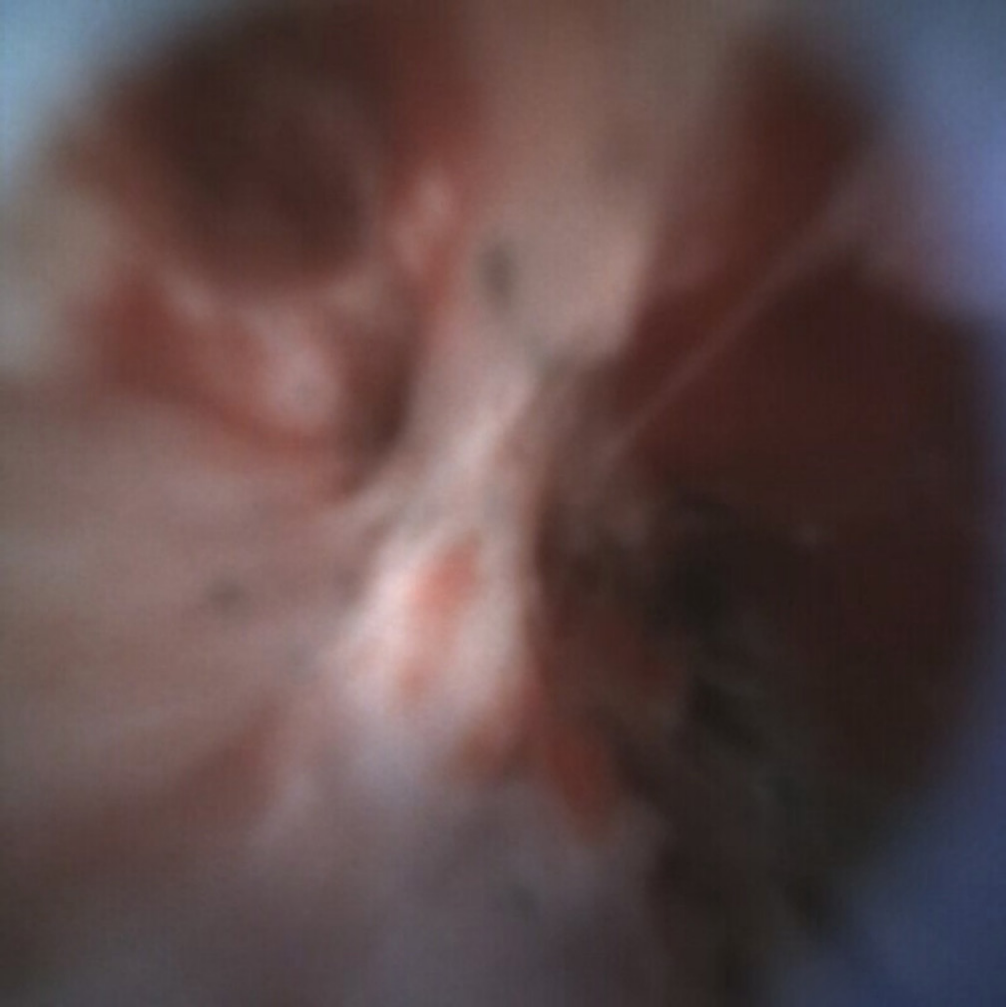
Altered Necrotic lymph node tissue with white discoloration.

**FIGURE 6:**
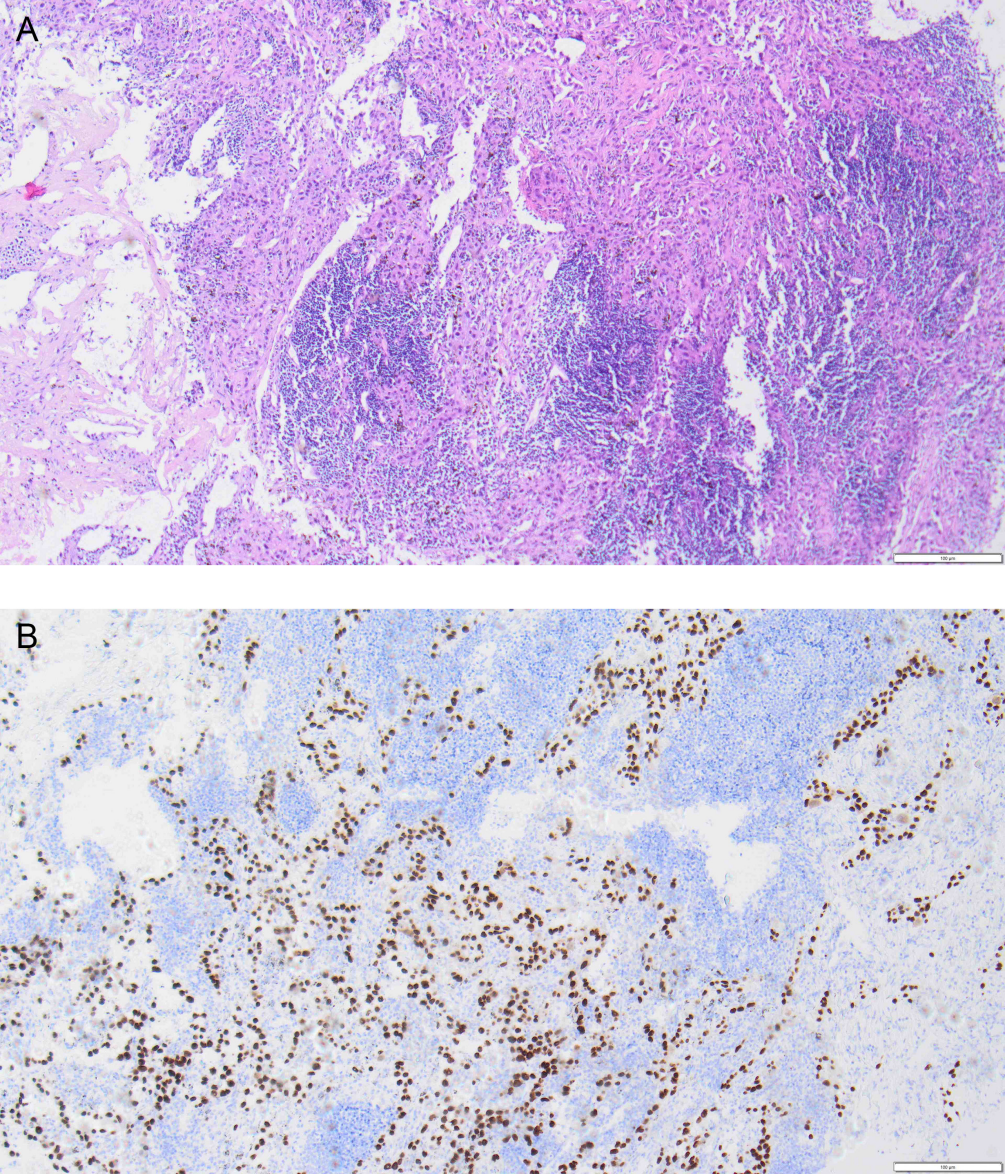
(A) and (B) HPE showing necrosis and presence of squamous cell carcinoma.

A 55‐year‐old non‐smoker with bilateral pulmonary nodules and suspected metastasis underwent EBUS for node assessment. The Iriscope was inserted post‐TBNA into 11L and 7, revealing presumed necrotic areas due to disrupted architecture and white discoloration in 11L (Figures 4 and 5). Station 7 presented with uniform reddish coloration. Histology showethe d presence of necrosis as well as squamous cell carcinoma in 11L nodes (Figure 6A,B), (Video 2), no malignancy was shown in the 7 nodes.

**VIDEO 2:** Iriscope guided intranodal lymph node visualisation for Case 3. Video content can be viewed at https://onlinelibrary.wiley.com/doi/10.1002/rcr2.70330.

### 2.4 Case 4

**FIGURE 7:**
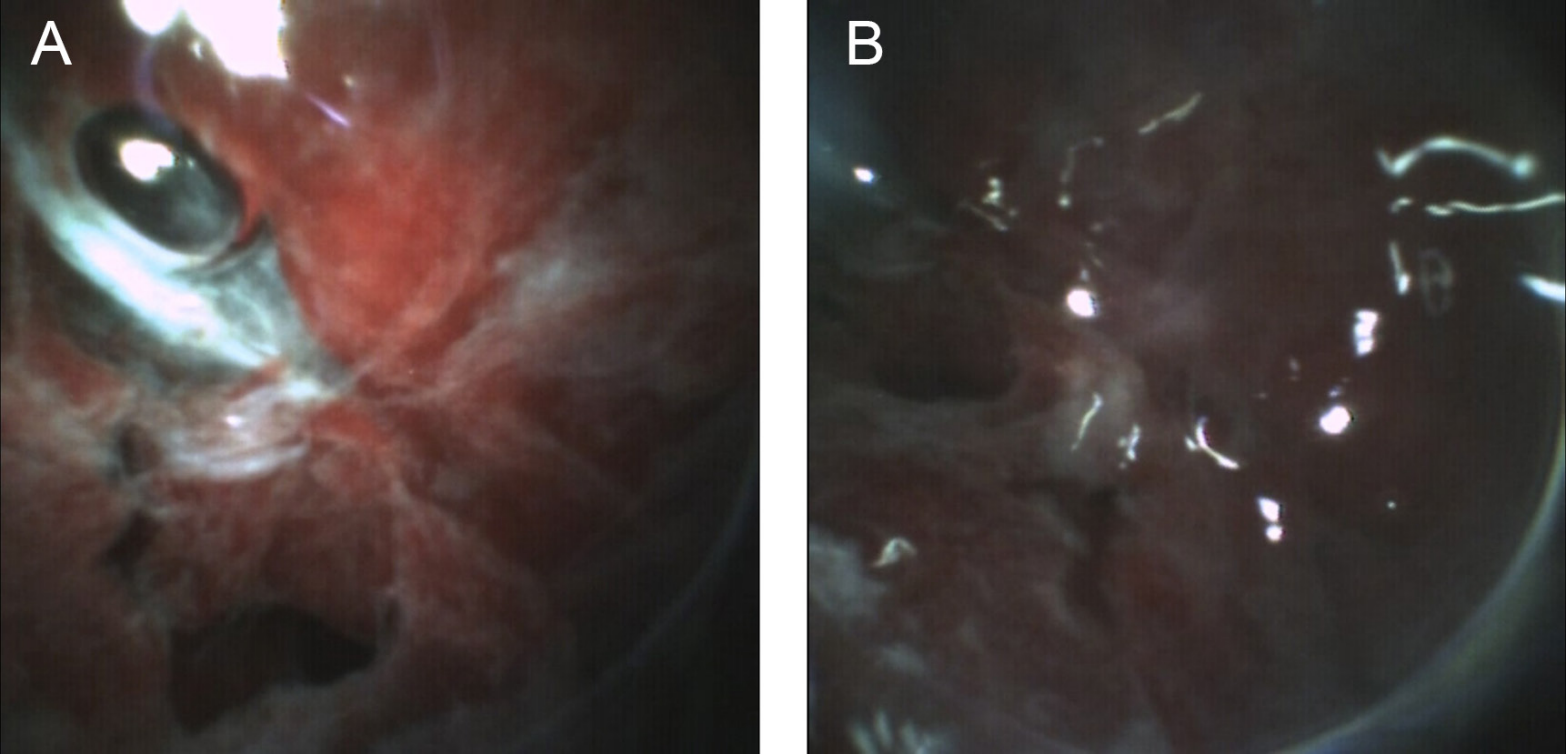
(A) and (B) Patchy white lesions observed in small cell carcinoma.

A 71‐year‐old male undergoing lung cancer staging was found to have enlarged 10R and 4R lymph nodes. EBUS‐assisted examination with the Iriscope was performed after TBNA and tract creation with the 22G crown‐tip needle. Intranodal video showed patchy white lesions and disrupted architecture in both stations (Figure 7A,B). Final pathology confirmed metastatic small cell lung carcinoma. The technique allowed targeted sampling of visually abnormal areas using forceps inserted through the guide sheath (Table 1).

**TABLE 1:** Summary of cases—iriscope findings and histopathological correlation.

Case	Station	Iriscope findings	Histopathological diagnosis
Case 1	4R	White nodular foci	Adenocarcinoma
	11R	Uniform red coloration	Benign lymph node
Case 2	7	Scattered dark grey pigmentation	Sacroidosis
Case 3	11L	Disrupted architecture and white discoloration	Necrotic squamous cell carcinoma
	7	Uniform red coloration	Benign lymph node
Case 4	4R and 10R	Patchy white lesions	Small cell lung carcinoma

## 3 Discussion

This case series represents a novel technique of direct intranodal imaging using the Iriscope during EBUS. The technique allows real‐time observation of lymph node morphology, potentially enhancing diagnostic yield by identifying visually suspicious areas. The average procedure time in our series was approximately 30–40 min, with the Iriscope exploration accounting for an average of 4 min per lymph node station. No complications were recorded in the cases.

Traditionally, EBUS‐TBNA has been limited by its reliance on indirect tissue sampling, where visual feedback is restricted to ultrasound imaging and macroscopic inspection of retrieved samples. The integration of miniature video endoscopy into the lymph node adds a real‐time, image‐guided layer that has the potential to improve targeting of suspicious regions and reduce false‐negative results, especially in heterogeneous or partially necrotic nodes.

In addition, the use of the Iriscope could support real‐time tissue characterisation. Preliminary observations from this series suggest that certain visual patterns—such as white discolouration, patchy grey discolouration, or uniform reddish tissue—may be associated with specific findings (e.g., carcinoma, granulomatous disease, or benign tissue). If validated in larger cohorts, these image‐based criteria could be used to triage biopsy sites, reduce unnecessary sampling, or even guide adjunctive diagnostic tools such as cryobiopsy or fine‐needle biopsy with ROSE (rapid on‐site evaluation).

Intranodal visualisation with Iriscope contrasts with other advanced imaging techniques that have been investigated for lymph node evaluation, such as optical coherence tomography (OCT) and confocal laser endomicroscopy (CLE). OCT and CLE typically provide grayscale, limited field‐of‐view images that require post‐processing and specialised interpretation. In contrast, the Iriscope delivers full‐colour, macroscopic video imaging, enabling real‐time visualisation of gross nodal architecture, discoloration patterns, and structural disruption, which can be immediately interpreted by the proceduralist. Importantly, the Iriscope is compatible with existing EBUS workflows and was deployed using standard guide sheaths without the need for additional platforms or equipment. This integration facilitates targeted sampling of visually suspicious areas and supports broader clinical feasibility. While future studies may explore complementary roles for CLE or OCT, our findings suggest that direct intranodal video endoscopy offers a practical and intuitive means of real‐time tissue assessment during EBUS‐TBNA.

However, the small sample size and observational nature preclude definitive conclusions about diagnostic accuracy or added clinical value. The interpretation of visual findings remains subjective and operator‐dependent, highlighting the need for standardised imaging criteria and training protocols. Additionally, this case series represents an early exploratory study. While the feasibility and technical success of intranodal videoendoscopy were consistently demonstrated, the case series was not designed or powered to assess comparative diagnostic accuracy, clinical outcomes, or impact on patient management. Without a control group or standardised reference test, the clinical utility of intranodal imaging remains uncertain. Also, the cost‐effectiveness and scalability of incorporating video endoscopy into routine EBUS workflows must be evaluated.

In conclusion, direct intranodal visualisation using the Iriscope is a feasible, safe, and promising technique that enriches the diagnostic landscape of mediastinal endoscopy. Further prospective studies are warranted to validate imaging criteria and assess its diagnostic impact as well as clinical utility.

## Author Contributions

All the authors contributed to the manuscript. The first draft of the manuscript was written by Sammy Onyancha, and all the authors commented on previous versions of the manuscript. All the authors have read and approved the final manuscript.

## Consent

The authors declare that written informed consent was obtained for the publication of this manuscript and accompanying images using the form provided by the Journal.

## Conflicts of Interest

The authors declare no conflicts of interest.

## Data Availability

The data that support the findings of this study are available from the corresponding author upon reasonable request.
